# The influence of the COVID-19 pandemic on surgical therapy and care: a cross-sectional study

**DOI:** 10.1186/s12893-022-01708-7

**Published:** 2022-07-05

**Authors:** Karl H. Hillebrandt, Simon Moosburner, Axel Winter, Nora Nevermann, Nathanael Raschzok, Thomas Malinka, Igor M. Sauer, Moritz Schmelzle, Johann Pratschke, Sascha Chopra

**Affiliations:** 1 Department of Surgery, Charité-Universitätsmedizin Berlin, Corporate Member of Freie Universität Berlin and Humboldt-Universität zu Berlin, Campus Charité Mitte | Campus Virchow-Klinikum, Augustenburger Platz 1, 13353 Berlin, Germany; 2 Berlin Institute of Health at Charité-Universitätsmedizin Berlin, BIH Academy, Clinician Scientist Program, Charitéplatz 1, 10117 Berlin, Germany

**Keywords:** COVID-19 pandemic, Surgery, Quality management

## Abstract

**Background:**

Due to the COVID-19 pandemic, an extensive reorganisation of healthcare resources was necessary—with a particular impact on surgical care across all disciplines. However, the direct and indirect consequences of this redistribution of resources on surgical therapy and care are largely unknown.

**Methods:**

We analysed our prospectively collected standardised digital quality management document for all surgical cases in 2020 and compared them to the years 2018 and 2019. Periods with high COVID-19 burdens were compared with the reference periods in 2018 and 2019.

**Results:**

From 2018 to 2020, 10,723 patients underwent surgical treatment at our centres. We observed a decrease in treated patients and a change in the overall patient health status. Patient age and length of hospital stay increased during the COVID-19 pandemic (p = 0.004 and p = 0.002). Furthermore, the distribution of indications for surgical treatment changed in favour of oncological cases and less elective cases such as hernia repairs (p < 0.001). Postoperative thromboembolic and pulmonary complications increased slightly during the COVID-19 pandemic. There were slight differences for postoperative overall complications according to *Clavien-Dindo*, with a significant increase of postoperative mortality (p = 0.01).

**Conclusion:**

During the COVID-19 pandemic we did not see an increase in the occurrence, or the severity of postoperative complications. Despite a slightly higher rate of mortality and specific complications being more prevalent, the biggest change was in indication for surgery, resulting in a higher proportion of older and sicker patients with corresponding comorbidities. Further research is warranted to analyse how this changed demographic will influence long-term patient care.

**Supplementary Information:**

The online version contains supplementary material available at 10.1186/s12893-022-01708-7.

## Background

The comprehensive medical burden of the COVID-19 pandemic has strained and, in some cases, exceeded the capacity of healthcare systems around the globe. Throughout the pandemic there has been a need for structural and personnel reorganisation of routine healthcare to support the treatment of patients suffering from a SARS-CoV-2 infection.

Especially surgical disciplines have suffered from the reduced capacities on intensive care units (ICU) and normal wards. To continue effective medical treatment, early during the crisis, several surgical societies published recommendations in case of capacity shortages due to local pandemic situations [1–6]*.* This included regional cooperation, guarantee of the supply chain, testing facilities, personal protective equipment and most importantly case prioritization and scheduling.

In a pan-European survey among surgical departments, most participants reported a high impact of the pandemic on surgical therapy i.e., suspended surgical procedures, restriction of capacities and a decrease in patient referral with suspected negative outcomes for the affected patients [7]. Moreover, the effect of a perioperative SARS-CoV-2 infection on the surgical outcome has recently been shown in a multicentre analysis by the *COVIDSurg* *Collaborative,* demonstrating an increase in pulmonary complications and 30-day mortality [8]. Furthermore, the *COVIDSurg* *Collaborative* observed that asymptomatic patients after a SARS-CoV-2 infection can be safely operated ≥ 7 weeks after diagnosis of the infection. Scheduled operations in symptomatic patients should be postponed longer to prevent an increased 30-day mortality [9]. Nevertheless, overall indication and timepoint for surgery have been delayed by the COVID-19 pandemic [10]. With outpatient screening programs reduced especially for colorectal and breast cancer in some countries and patients therefore presenting with a progressed disease stage [11, 12]. Furthermore, the focus of the health care systems in most countries towards patients of older age with COVID-19, has had a detrimental effect on the care for routine surgical procedures such as hip fractures [13]

However, the influence of this structural and personnel reorganisation on the quality of surgical therapy and postoperative care needs to be further investigated. Data especially on complications after surgical therapy remain scarce. We hypothesized, that due to the COVID-19 pandemic we would see an increase in the occurrence of postoperative complications in addition to a higher severity of complications as measured by *Clavien-Dindo*.

Therefore, we wanted to assess the effect of the COVID-19 pandemic on postoperative complications, in a large tertiary referral centre. To determine the effect of the COVID-19 pandemic we used our previously established digital quality management (QM) documentation system, which is used in daily routine [14]. For each patient discharged from our department a QM document is created and reviewed on the day after discharge by a board of consultant surgeons.

## Methods

### Study design

In this observational cross-sectional study, all patients admitted to the Department of Surgery, Campus Charité Mitte and Campus Virchow Klinikum, Charité-Universitätsmedizin Berlin, Germany, for surgical treatment and care from January 1st 2018 to December 31st 2020 with complete QM documents (Additional file 1: Fig. S1) were included. QM documents were introduced as of late 2017 in our clinic. The QM document is prospectively collected on the day of discharge and validated daily by a group of senior consultant surgeons. It includes the indication for surgical treatment, complications which occurred during the in-patient stay and the necessary procedure for complication management. Complications are classified either as medical or organizational. Medical complications are further classified into renal, post pancreatic surgery, central nervous system, post vascular surgery, cardiovascular, systemic, or surgical site infections, bleeding, post organ transplantation, pulmonary, gastrointestinal, and hepatobiliary complications. Finally, complications are classified according to *Clavien-Dindo* [15]. Additionally, complication management, i.e. surgery, intensive care unit, percutaneous drainage, dialysis, angiography, gastric tube, chest tube, transfusion or cardiopulmonary resuscitation is documented. Patients were not followed up after discharge. Primary endpoint of this study were the frequency of postoperative complications during COVID-19 periods vs. non-COVID-19 periods.

**Fig. 1:**
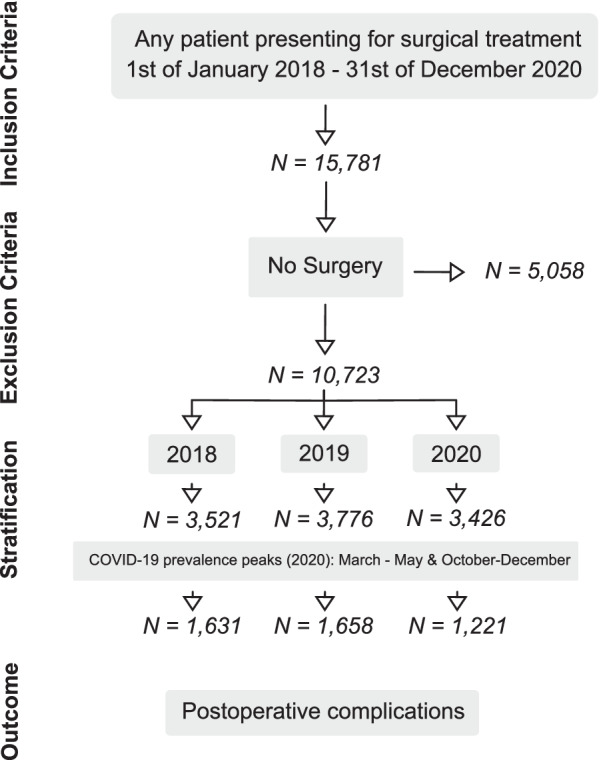
Patient flow chart, inclusion and exclusion criteria. Patients were stratified after year of surgical treatment (2018, 2019 and 2020) as well as the corresponding time periods of COVID-19 prevalence peaks (March–May and October–December of 2020)

We defined two COVID-19 periods for 2020 with the highest COVID-19 burdens i.e., increased rates of new SARS-CoV-2-infections in Germany (Fig. 1) [16]. The first period was from 1st of March 2020 to 1st of May 2020 (COVID-19-1) and the second period from 1st of October 2020 to 31st of December 2020 (COVID-19-2). Both these high-incidence *COVID-19* *periods* (COVID-19) combined were compared with the same periods in 2019 and 2018 (*reference* *periods* [REF-2018 and REF-2019]). Moreover, we compared the complete *COVID-19-year* 2020 against the years 2018 and 2019.

As the distribution of indication for surgery and indeed patient operated on itself were secondary outcome parameters, a sample size calculation would have artificially ended the study at a given time.

In addition to the QM document, the American Society of Anaesthesiologist (ASA) Score and the German procedure classification (*Operationen-* *und* *Prozedurenschlüssel*, OPS) codes were extracted from the patient data management system (SAP SE, Walldorf, Germany). Major and minor surgical procedures were defined according to Baum et al*.* [17].

### Ethical approval

The ethics committee of the Charité-Universitätsmedizin Berlin approved the study (ethical approval code: EA1/149/21). The need for informed consent was waived by the institutional ethics comittee due to the nature of this study. Investigational methods used in this study were implemented in accordance with the relevant guidelines and regulations of the ethics committee.

### Statistical analysis

Data was analysed using *R* (version 4.0.5) and *RStudio* (version 1.4) for macOS (R Foundation for Statistical Computing, Vienna, Austria). Packages for statistical analysis and graph plotting included tidyverse, gtsummary, ggpubr, forestmangr, broom, scales and rcompanion. Overall, a two-sided p-value < 0.05 was considered statistically significant. Missing data was excluded from analysis. Categorical variables were compared using the Pearson's chi-squared or Fisher's exact test. Continuous variables were analysed using the Kruskal–Wallis test. Data are reported as counts and percentages or median and interquartile range (IQR). Results were adjusted for multiple testing using the method proposed by Benjamini and Hochberg and are reported as q values. A positive false discovery rate (q-value) cut-off of ≤ 0.05 was chosen.

To account for potential confounding factors, multivariable linear Regression was performed for length of hospital stay and binomial logistic regression for mortality after surgical intervention. Results are reported as odds ratio (OR) with 95% confidence interval for logistic regression (CI) and estimates of the beta coefficients for linear regression. Included variables for both regression models were age, sex, length of hospital stay, year, clinic campus, indication for surgery, ASA-score, COVID-19 period and complications classified by *Clavien-Dindo*. Explained variability is reported as Nagelkerke pseudo R-squared for both models.

## Results

### Patient and procedure demographics

**Fig. 2:**
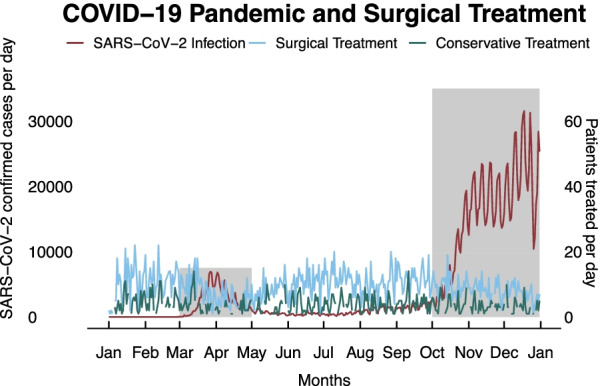
SARS-CoV-2 Infections in Germany 2020. Daily rate of new infections as reported by the Robert-Koch-Institute. Periods of high COVID-19 burden (COVID-19 Period; COVID-19) from March to May and October to end of December 2020 are highlighted in red. Number of patients surgically treated at our centre in blue, patients receiving conservative treatment in green

Between January 2018 and December of 2020 there were a total of 15,781 cases on both campi of our department. Of those patients, 67.9% (n = 10,782) received operative treatment and were included in further analysis (Fig. 1). We observed a 32.6% decrease of treated patients for the COVID-19 period as well as a 14.7% decrease for 2020 compared to the previous years. Overall, there were more cases of operative treatment during the COVID-19 period (73% vs. 70% and 66% in REF-2019 and REF-2018) and 2020 (2020/74% vs. 66% and 60% in 2019 and 2018)) (Fig. 2).

**Fig. 3:**
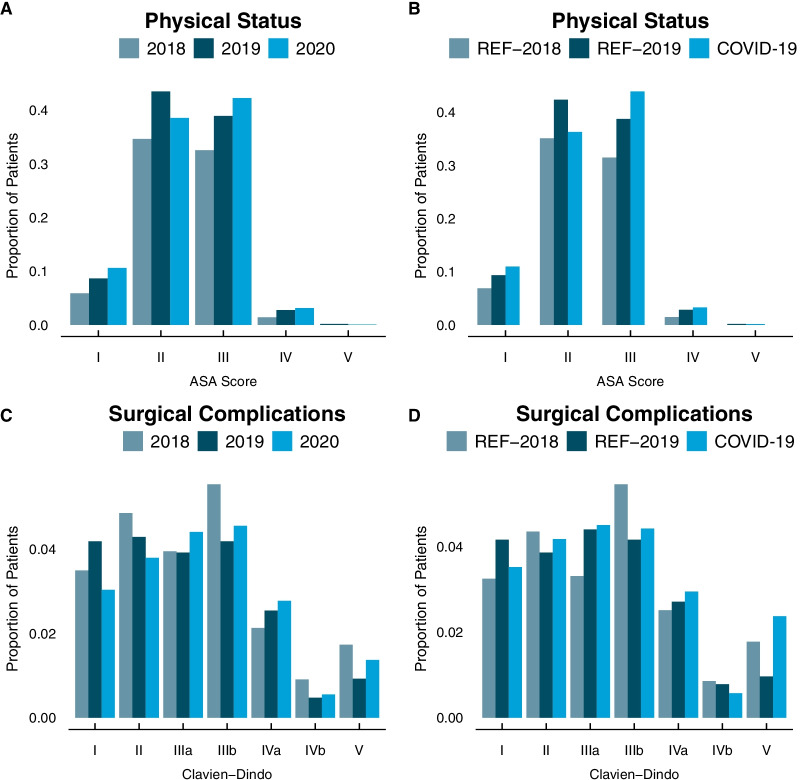
Physical status according to the ASA-Score and complications classified after Clavien-Dindo. Patients are stratified after analysis year as well as periods of high COVID-19 burden (COVID-19) from March to May and October to end of December 2020 compared to the corresponding reference (REF) period in 2018 and 2019. **A**, **B** ASA-Score. **C**, **D** Complications

We observed a significant towards higher ASA-Scores for patients operated during the COVID-19 period and 2020 (both p < 0.001, Fig. 3; Table 1).

**Table 1:** Descriptive patient data

Variable	Year						COVID-19 period					
	Overall, N = 10,723^a^	2018, N = 3521^a^	2019, N = 3776^a^	2020, N = 3426^a^	p-value^b^	q-value^c^	Overall, N = 4510^a^	2018 Reference, N = 1631^a^	2019 Reference, N = 1658^a^	COVID-19, N = 1221^a^	p-value^b^	q-value^c^
Sex (f)	4744 (44%)	1517 (43%)	1688 (45%)	1539 (45%)	0.2	0.3	1990 (44%)	705 (43%)	749 (45%)	536 (44%)	0.5	0.6
Age in years	52 (38, 63)	53 (39, 64)	52 (37, 63)	52 (38, 63)	0.068	0.11	52 (38, 63)	52 (38, 64)	52 (37, 63)	54 (40, 64)	0.038	0.10
Campus					< 0.001	0.002					0.5	0.5
Charite Mitte	4166 (39%)	1459 (41%)	1407 (37%)	1300 (38%)			1671 (37%)	621 (38%)	612 (37%)	438 (36%)		
Charite Virchow-Klinikum	6557 (61%)	2062 (59%)	2369 (63%)	2126 (62%)			2839 (63%)	1.10 (62%)	1.46 (63%)	783 (64%)		
Length of hospital stay	7 (5, 13)	8 (5, 14)	7 (5, 12)	7 (5, 13)	< 0.001	0.001	8 (5, 14)	8 (5, 13)	7 (5, 13)	8 (5, 15)	0.002	0.013
Indication for surgery					< 0.001	< 0.001					< 0.001	< 0.001
Appendix	542 (5.1%)	135 (3.8%)	213 (5.6%)	194 (5.7%)			231 (5.1%)	77 (4.7%)	88 (5.3%)	66 (5.4%)		
Colorectal	1,715 (16%)	513 (15%)	608 (16%)	594 (17%)			753 (17%)	239 (15%)	278 (17%)	236 (19%)		
Gallbladder	485 (4.5%)	133 (3.8%)	192 (5.1%)	160 (4.7%)			213 (4.7%)	82 (5.0%)	83 (5.0%)	48 (3.9%)		
Hepatobiliary	916 (8.5%)	317 (9.0%)	318 (8.4%)	281 (8.2%)			407 (9.0%)	149 (9.1%)	144 (8.7%)	114 (9.3%)		
Hernia	963 (9.0%)	339 (9.6%)	368 (9.7%)	256 (7.5%)			387 (8.6%)	152 (9.3%)	167 (10%)	68 (5.6%)		
Others	1,111 (10%)	352 (10.0%)	414 (11%)	345 (10%)			414 (9.2%)	144 (8.8%)	163 (9.8%)	107 (8.8%)		
Pancreas	554 (5.2%)	179 (5.1%)	200 (5.3%)	175 (5.1%)			216 (4.8%)	74 (4.5%)	80 (4.8%)	62 (5.1%)		
Parathyroid	213 (2.0%)	63 (1.8%)	86 (2.3%)	64 (1.9%)			82 (1.8%)	30 (1.8%)	32 (1.9%)	20 (1.6%)		
Peritoneal malignancies	192 (1.8%)	74 (2.1%)	62 (1.6%)	56 (1.6%)			75 (1.7%)	34 (2.1%)	23 (1.4%)	18 (1.5%)		
Small intestine	408 (3.8%)	140 (4.0%)	122 (3.2%)	146 (4.3%)			182 (4.0%)	67 (4.1%)	60 (3.6%)	55 (4.5%)		
Thorax	1,362 (13%)	455 (13%)	438 (12%)	469 (14%)			576 (13%)	218 (13%)	185 (11%)	173 (14%)		
Thyroid	536 (5.0%)	152 (4.3%)	194 (5.1%)	190 (5.5%)			214 (4.7%)	59 (3.6%)	91 (5.5%)	64 (5.2%)		
Transplantation	466 (4.3%)	152 (4.3%)	148 (3.9%)	166 (4.8%)			193 (4.3%)	61 (3.7%)	72 (4.3%)	60 (4.9%)		
Upper GI	842 (7.9%)	240 (6.8%)	295 (7.8%)	307 (9.0%)			389 (8.6%)	114 (7.0%)	152 (9.2%)	123 (10%)		
Vascular	418 (3.9%)	277 (7.9%)	118 (3.1%)	23 (0.7%)			178 (3.9%)	131 (8.0%)	40 (2.4%)	7 (0.6%)		
Postoperative complications					< 0.001	0.002					0.2	0.3
No complications	8450 (79%)	2,725 (77%)	3001 (79%)	2724 (80%)			3535 (78%)	1280 (78%)	1309 (79%)	946 (77%)		
Clavien-Dindo I	385 (3.6%)	123 (3.5%)	158 (4.2%)	104 (3.0%)			165 (3.7%)	53 (3.2%)	69 (4.2%)	43 (3.5%)		
Clavien-Dindo II	463 (4.3%)	171 (4.9%)	162 (4.3%)	130 (3.8%)			186 (4.1%)	71 (4.4%)	64 (3.9%)	51 (4.2%)		
Clavien-Dindo IIIa	438 (4.1%)	139 (3.9%)	148 (3.9%)	151 (4.4%)			182 (4.0%)	54 (3.3%)	73 (4.4%)	55 (4.5%)		
Clavien-Dindo IIIb	509 (4.7%)	195 (5.5%)	158 (4.2%)	156 (4.6%)			212 (4.7%)	89 (5.5%)	69 (4.2%)	54 (4.4%)		
Clavien-Dindo IVa	266 (2.5%)	75 (2.1%)	96 (2.5%)	95 (2.8%)			122 (2.7%)	41 (2.5%)	45 (2.7%)	36 (2.9%)		
Clavien-Dindo IVb	69 (0.6%)	32 (0.9%)	18 (0.5%)	19 (0.6%)			34 (0.8%)	14 (0.9%)	13 (0.8%)	7 (0.6%)		
Clavien-Dindo V	143 (1.3%)	61 (1.7%)	35 (0.9%)	47 (1.4%)			74 (1.6%)	29 (1.8%)	16 (1.0%)	29 (2.4%)		
ASA					< 0.001	0.002					< 0.001	0.006
ASA 1	898 (9.5%)	207 (7.9%)	327 (9.2%)	364 (11%)			401 (10%)	112 (9.2%)	155 (10%)	134 (12%)		
ASA 2	4184 (44%)	1220 (47%)	1643 (46%)	1321 (41%)			1,717 (44%)	572 (47%)	702 (45%)	443 (38%)		
ASA 3	4064 (43%)	1146 (44%)	1470 (41%)	1448 (45%)			1,691 (43%)	513 (42%)	642 (41%)	536 (46%)		
ASA 4	261 (2.8%)	50 (1.9%)	104 (2.9%)	107 (3.3%)			111 (2.8%)	24 (2.0%)	47 (3.0%)	40 (3.5%)		
ASA 5	9 (< 0.1%)	0 (0%)	7 (0.2%)	2 (< 0.1%)			5 (0.1%)	0 (0%)	3 (0.2%)	2 (0.2%)		
Type of surgery					0.006	0.016					0.5	0.5
Minor resection	7250 (68%)	2452 (70%)	2505 (66%)	2293 (67%)			3031 (67%)	1114 (68%)	1107 (67%)	810 (66%)		
Major resection	3473 (32%)	1069 (30%)	1271 (34%)	1133 (33%)			1479 (33%)	517 (32%)	551 (33%)	411 (34%)		
Complication												
Bleeding	294 (2.7%)	102 (2.9%)	93 (2.5%)	99 (2.9%)	0.4	0.5	117 (2.6%)	41 (2.5%)	33 (2.0%)	43 (3.5%)	0.037	0.10
Gastrointestinal	543 (5.1%)	180 (5.1%)	181 (4.8%)	182 (5.3%)	0.6	0.7	234 (5.2%)	74 (4.5%)	82 (4.9%)	78 (6.4%)	0.075	0.2
Cardiovascular	207 (1.9%)	82 (2.3%)	63 (1.7%)	62 (1.8%)	0.10	0.2	95 (2.1%)	37 (2.3%)	28 (1.7%)	30 (2.5%)	0.3	0.5
Hepatobiliary	216 (2.0%)	74 (2.1%)	78 (2.1%)	64 (1.9%)	0.8	0.8	96 (2.1%)	27 (1.7%)	39 (2.4%)	30 (2.5%)	0.2	0.4
Organisational	384 (3.6%)	231 (6.6%)	87 (2.3%)	66 (1.9%)	< 0.001	< 0.001	189 (4.2%)	116 (7.1%)	34 (2.1%)	39 (3.2%)	< 0.001	< 0.001
Pancreatic	130 (1.2%)	42 (1.2%)	53 (1.4%)	35 (1.0%)	0.3	0.4	63 (1.4%)	19 (1.2%)	28 (1.7%)	16 (1.3%)	0.4	0.5
Pulmonary	741 (6.9%)	269 (7.6%)	235 (6.2%)	237 (6.9%)	0.058	0.11	336 (7.5%)	124 (7.6%)	112 (6.8%)	100 (8.2%)	0.3	0.5
Renal	453 (4.2%)	174 (4.9%)	157 (4.2%)	122 (3.6%)	0.016	0.036	199 (4.4%)	74 (4.5%)	69 (4.2%)	56 (4.6%)	0.8	0.8
Infectious	281 (2.6%)	120 (3.4%)	77 (2.0%)	84 (2.5%)	< 0.001	0.003	123 (2.7%)	47 (2.9%)	36 (2.2%)	40 (3.3%)	0.2	0.3
Transplantation	142 (1.3%)	44 (1.2%)	38 (1.0%)	60 (1.8%)	0.020	0.039	60 (1.3%)	16 (1.0%)	18 (1.1%)	26 (2.1%)	0.016	0.074
Surgical site infection	604 (5.6%)	196 (5.6%)	207 (5.5%)	201 (5.9%)	0.8	0.8	253 (5.6%)	75 (4.6%)	93 (5.6%)	85 (7.0%)	0.025	0.091

*LOS* length of hospital stay, *GI* gastrointestinal, *ASA* American Society of Anesthesiologists, *SSI* surgical site infection

^a^n (%); Median (IQR)

^b^Pearson's Chi-squared test; Kruskal–Wallis rank sum test

^c^False discovery rate correction for multiple testing

**Fig. 4:**
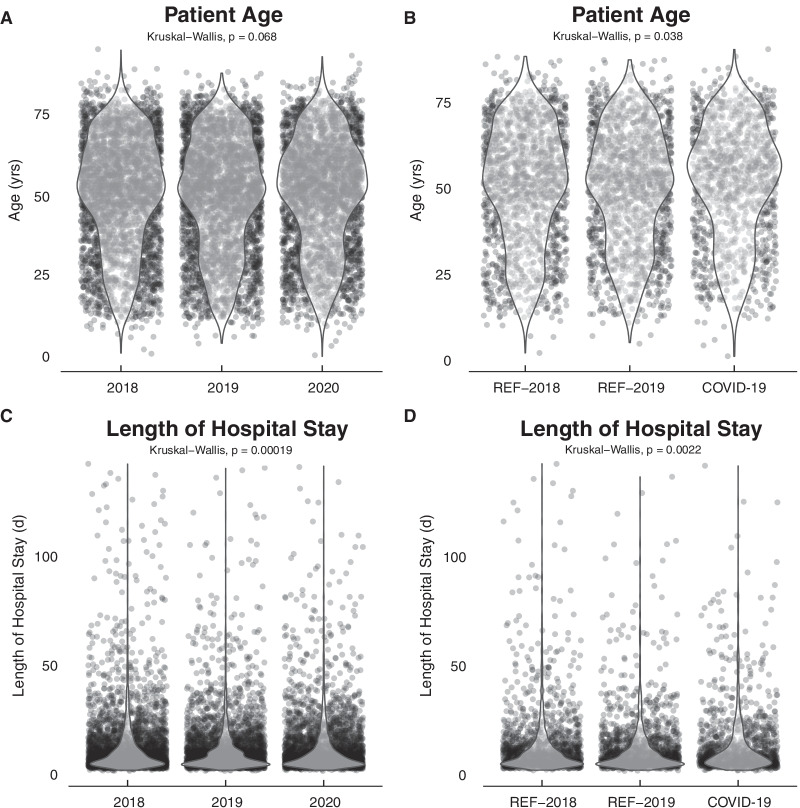
Patient demographics stratified after analysis year as well as periods of high COVID-19 burden (COVID-19) from March to May and October to end of December 2020 compared to the corresponding reference (REF) period in 2018 and 2019. **A**, **B** Length of hospital stay in days 2019. **B**–**D** Patient age in years

Furthermore, we observed a decrease of hernia procedures for COVID-19 and 2020 2020: 7.5%; 2019: 9.7%; 2018: 9.6% and COVID-19: 5.6%, REF-2019: 10% and REF-2018: 9.3%, respectively). Although there was a trend for an increase of hepato-biliary, pancreatic, colorectal, and upper-GI procedures, none showed statistically significant changes. Major and minor procedures were more prevalent in 2019 and 2020 (34% and 33%) compared to 2018 (30%). Further the COVID 19-period there was no difference compared to the reference periods in the previous years. Median length of hospital stay varied in between analysis years as well as the COVID-19 period compared to the reference periods (p < 0.001 and p = 0.002, respectively) (Fig. 4). Median length of hospital stay was one day longer (8 days) than REF-2019 (7 days), however the same as REF-2018 (8 days).

In our linear regression model for length of hospital stay (Beta zero (intercept) = 12.0), which explained 49.8% of variance (Nagelkerke pseudo R-squared = 0.480,), The COVID-19 period was not an independent factor significantly associated with a prolonged length of hospital stay (Beta = − 0.4, CI − 1.3 to 0.47; p = 0.4,). With increasing grade of complication after Clavien-Dindo, length of hospital stay increased (Clavien-Dindo IVb; Beta = 54, CI 50–58; p < 0.001). Dependent on the indication for surgical treatment, hospital stay was either longer, i.e. in cases of organ transplantation (Beta = 6.3, CI 4.3–8.2; p < 0.001) or shorter for hernia repair (Beta = − 2.3, CI − 3.8 to − 0.7; p = 0.004). Sex and age of the patient did not have an influence on length of hospital stay (both p = 0.9).

### Clavien-Dindo classification

For the complications according to Clavien-Dindo in general there was a difference in distribution comparing years and the COVID-19 period to the reference periods, however with no clear trend. Exclusively analysing postoperative mortality (Clavien-Dindo V) we saw a significant decrease comparing 2019 to 2018 (0.9% vs. 1.7%, p = 0.01), with only a slight increase in 2020 (1.4% vs. 0.9%, p = 0.26). The COVID-19 period had the highest mortality with 2.4% compared to 1% in REF-2019 and 1.8% in REF-2018. Mortality only differed between REF-2019 and COVID-19 (p = 0.01).

In our binomial logistic regression model for postoperative mortality which explained 37% of variance (Nagelkerke pseudo R-squared = 0.3367), The COVID-19 period was not an independent risk factor (OR 0.13 CI − 0.52 to 0.78, p = 0.7,). Risk factors explaining postoperative mortality were a higher ASA score (ASA 5, OR 6.4 CI 4–9, p < 0.001) and a higher patient age (OR 0.05 CI 0.03–0.08, p < 0.001).

### Specific complications: gastrointestinal

During the COVID-19 period there were significantly more anastomotic leakages (1.7%, REF-2019: 0.7%, REF-2018: 0.6%, p = 0.005) as well as in 2020 (1.4%, 2019:0.7%, 2018:1.1%) even when correcting for multiple testing (0.p = 0.03 and q = 0.04). Overall, patients with a higher ASA-score had more anastomotic leakages (p < 0.001).

### Specific complications: cardiovascular and pulmonary

Although overall cardiovascular complications remained equally distributed, during the COVID-19 period, we recorded a slight increase of venous thrombosis (0.7%, REF-2019: 0.1%, REF-2018: 0.3%, p = 0.05; q = 0.2), without the same effect over the years 2018–2020 (p = 02). In line with these findings, we also observed an increase in pulmonary artery embolism for the COVID-19 period (1.1%, REF-2019% REF-2018 0.4%, p = 0.03, q = 0.1). During the COVID-19 period more patients developed pneumonia during the postoperative course (p = 0.02, q = 0.1). For the COVID-19 period and the year 2020 there was an increase in unplanned reintubation. Reintubation and pulmonary artery embolism were significantly increased in patients with a higher ASA-Score (p < 0.001 for both parameters).

### Specific complications—transplantation

For the COVID-19 period and 2020, more rejections were documented during the postoperative course after organ transplantation (COVID-19 p = 0.009 and 2020 p = 0.045, q = 0.3). Furthermore, we observed a significant increase of delayed graft functions for 2020 (p < 0.001). For all other transplant associated complications no significant differences could be identified.

### Specific complications: others

For all other documented complications (Additional file 1: Fig. S1), no significant differences were found for either 2020 or the COVID-19 period. Especially, for HPB complications we could not observe a change during COVID-19 although the HPB procedures increased during the COVID-19 (Table 1).

### Complication management

The QM documents also record the management of the complications. In the COVID-19 period and 2020, we observed that significantly more patients received postoperative blood transfusions compared to the reference periods and years (p = 0.047 and p = 0.011). Additionally, more percutaneous drainages were place (COVID-19 p = 0.02 and 2020 p = 0.03).

## Discussion

To our knowledge this is one of the first studies which specifically analysed the overall impact of the COVID-19 pandemic on postoperative complications. We hypothesized, that due to the COVID-19 pandemic we would see an increase in the occurrence and the severity of postoperative complications. This was not the case. Several specific complications such as transplantation, gastrointestinal or pulmonary complications were more prevalent during the COVID-19 periods, however, the biggest change was in indication for surgery. As recommended by most surgical societies, non-urgent procedures, e.g. hernia surgeries, were cancelled or postponed, resulting in a significant decrease in numbers. The change in procedures and therefore in the patient spectrum is most likely the reason for the significant increase of both, the median length of hospital stay and patient age during the COVID-19 period. This suggestion is in line with fewer hernia procedures and the relative increase of major resections as well as the increase of, for example, upper-GI or colorectal procedures. Comparable results for median patient age and length of hospital stay were also observed for patients with emergency general surgery admissions in the *United* *Kingdom* during the first COVID-19 pandemic period [18].

Particularly interesting is the increase of thromboembolic and pulmonary events especially during the COVID-19 periods. Despite systemic inflammatory response processes being associated with postoperative thromboembolic complications, we hypothesize nevertheless that these findings might, in part, be related to the hygiene concepts to prevent in-hospital SARS-CoV-2 infections (e.g., visiting ban). These restrictions may have led to a reduced mobility and therefore an increase of thromboembolic and pulmonary complications. How important the postoperative mobilisation and the positive effect of the family implementation can be, has been shown previously [19–21]. Nevertheless, we lack in-depth data on preoperative medication and pre-existing conditions increasing the likelihood of thromboembolic complications. Moreover, patients with higher ASA-Scores had more unplanned re-intubations and pulmonary artery embolisms. It has been shown that a higher ASA-Score is a risk factor for pulmonary artery embolisms and deep vein thrombosis. From our point of view, this fact, and the increased age of our cohort and the shift towards more severe surgical cases, are the main reasons for the increase rate of venous thromboembolisms [22, 23].

One of the most concerning observations of our analysis is the significant increase of postoperative mortality during COVID-19 in univariate analysis. In our binominal logistic regression model, the strongest predictor remained the preoperative ASA score. The evaluation of the ASA score showed that the patients during COVID-19 and 2020 had significant higher ASA scores. It is well established, that the ASA score is a predictor for postoperative outcome with a significant correlation between the ASA score and postoperative complications and mortality [24]. From our point of view this shift to patients with a higher ASA score during COVID-19 period might be the main cause of the slightly increased mortality. Moreover, our department is the largest referral surgical unit in the Berlin-Brandenburg metropolitan area and therefore an assembly point for the most complex surgical cases. It is possible that this situation might have been aggravated during COVID-19 period due to capacity reduction in other hospitals and contributed to the increased postoperative mortality. However, this hypothesis needs to be proven in further investigations.

McLean et al*.* analysed similar parameters to ours in a single centre observational study for patients with general emergency surgery. They observed an increase in the *Charlson* *Comorbidity* *Index* and a change in the ASA score distribution albeit not reaching statistical significance. Moreover, they observed a significant increase in all-cause mortality [18]. Another important finding is the increase in unplanned stoma formation and may be related to the lockdown restrictions and resulting delay in medical presentation. On the other hand, this finding conforms with the results from the *COVID-19Surg* *Collaborative* on elective colorectal surgery. It was observed that there was a more frequent stoma formation compared to the pre-pandemic era [25].

The transferability of these findings is likely limited since our QM document only recognizes unplanned stoma formation. However, we hypothesized the underlying background to be the same. The surgeon responsible for the stoma formation aims to avoid postoperative complications which might go hand in hand with a need for ICU stay or prolonged length of hospital stay. Moreover, we found during the COVID-19 period a significant higher rate of postoperative bleedings compared to the reference period. Again, this is most likely associated with the shift in the patient collective towards patients with a higher ASA-score. This hypothesis might be supported by a study published by Lock et al*.* where they analysed postoperative bleeding complications in patients with perioperative anticoagulation bridging vs. patients without bridging. The colleagues observed a higher postoperative bleeding rate in patients with a bridging therapy compared to the control group. More interestingly, significantly more patients in the bridging group had an ASA-score > 2 compared to the control group [26].

Another interesting finding is the increase of gastrointestinal anastomotic leakages during 2020 and COVID-19. In general, in our sample, more anastomotic leakage occurred in patients with a higher ASA score. Considering that during COVID-19 and 2020 the ASA-Score distribution of our patients significantly changed towards patients with a higher ASA-Score, this situation might have contributed to the observed increase of anastomotic leakages.

Since the beginning of COVID-19 pandemic, the impact on solid organ transplantation was apparent. Due to the structural reorganizations caused by the pandemic, there was decrease of regional transplant activities during the first wave. Moreover, patients after solid organ transplantation represent a vulnerable collective, caused by the immunosuppression. Danziger-Isakov et al*.* raised the concern that due to an administration of less potent immunosuppressive drugs during the pandemic, there could be an increase of acute rejection rates [27]. Only selected studies reported their postoperative rejection rates after solid organ transplantation [27]. They did not observe increased rejection rates compared to the pre COVID-19 era. On the other hand, there was a significant increase of rejection for both 2020 and the COVID-19 period compared to the reference periods in our cohort. However, further studies are urgently needed to assess postoperative rejection rates during the COVID-19 pandemic. The reasons for our observation will be further investigated on a case-by-case basis to identify possible risk factors for rejections and if indeed a causality to the COVID-19 pandemic exists. Nevertheless, our observational study provides a comprehensive insight into the pandemic-related consequences for the surgical therapy and care in a tertiary referral centre. If our findings are either centre-specific with regional aspects or general indirect COVID-19-associated effects, requires further evaluation.

## Limitations

This study is limited by its single-centre design, and lack of more in-depth patient data. Although the QM documents are prospectively collected and reviewed by a board of consultant surgeons, the quality of the data may vary. Moreover, the QM documents are secondary data and thereby more likely to be prone to error. And quite interesting in this case, the QM document does not collect data regarding a perioperative SARS-CoV-2 infection. Due to the fact, that the QM document was introduced during late 2017, we could not evaluate QM data prior to 2018.

Finally, if patients are transferred within the hospital and discharged from another discipline, a QM document is not necessarily generated. Nevertheless, this is a rare situation and to date we are unaware of a similar QM document, systematically collected, that has been previously published.

## Conclusion

We hypothesized, that due to the COVID-19 pandemic we would see an increase in the occurrence of postoperative complications in addition to a higher severity of complications as measured by Clavien-Dindo. This was not the case. Despite a slightly higher rate of mortality and specific complications being more prevalent, the biggest change was in indication for surgery, resulting in a higher proportion of older and sicker patients with corresponding comorbidities. Adapting to this changed demographic worked in our tertiary care centre with abundant facilities for complication management, further research in smaller clinics is however definitely required.

## Supplementary Information

**Additional file 1**: **Figure S1**. Englisch translation of digital quality management document. QM documents are prospectively collected on the day of discharge and validated daily by a group of senior consultant surgeons. It includes the indication for surgical treatment (blue), complications which occurred during the inpatient stay (red) and the necessary procedure for complication management (green). Complications are classified either as medical or organizational. Medical complications are further classified into renal, post pancreatic surgery, central nervous system, post vascular surgery, cardiovascular, systemic, or surgical site infections, bleeding, post organ transplantation, pulmonary, gastrointestinal, and hepatobiliary complications. Finally, complications are classified according to Clavien-Dindo.

## Data Availability

The datasets used and/or analyzed during the current study are available from the corresponding author on reasonable request.
