# Supplementary material for: The influence of the COVID-19 pandemic on surgical therapy and care: a cross-sectional study
Source: BMC Surg. 2022 Jul 5;22:259. doi: 10.1186/s12893-022-01708-7 (PMC9253238; doi:10.1186/s12893-022-01708-7)
Supplement: Supplementary file 1 — Additional file 1: Figure S1. Englisch translation of digital quality management document. QM documents are prospectively collected on the day of discharge and validated daily by a group of senior consultant surgeons. It includes the indication for surgical treatment (blue), complications which occurred during the inpatient stay (red) and the necessary procedure for complication management (green). Complications are classified either as medical or organizational. Medical complications are further classified into renal, post pancreatic surgery, central nervous system, post vascular surgery, cardiovascular, systemic, or surgical site infections, bleeding, post organ transplantation, pulmonary, gastrointestinal, and hepatobiliary complications. Finally, complications are classified according to Clavien-Dindo. [file 12893_2022_1708_MOESM1_ESM.pdf]

Supplementary Material to:

The Influence of the COVID-19 Pandemic on Surgical Therapy and Care: a Cross-sectional Study

Indication

Upper GI	☐ malignant	☐ benign
Small intestine	☐ malignant	☐ benign
Colorectal	☐ malignant	☐ benign
Appendix	☐ malignant	☐ benign
Thyroid	☐ malignant	☐ benign
Parathyroid	☐ malignant	☐ benign
Pancreas	☐ malignant	☐ benign
Hepatobiliary	☐ malignant	☐ benign
Gallbladder	☐ malignant	☐ benign
Kidney	☐ malignant	☐ benign
Thorax	☐ malignant	☐ benign

☐ Bariatric
☐ Visualization of percutaneous drainage

Hernia
☐ Inguinal
☐ Umbilical
☐ Incisional

Transplantation
☐ Current Transplantation
☐ Kidney
☐ Liver
☐ Pancreas
☐ Small bowel
☐ Multivisceral
☐ Check-up
☐ ERCP/PTCD

☐ Vascular
☐ Other
☐ Sarcoma
☐ Peritoneal carcinomatosis

Renal complications

☐ Ureteral leak
☐ Urinoma
☐ Electrolyte imbalance
☐ Unplanned dialysis
☐ Acute kidney injury
☐ Intravenous fluid therapy
☐ Urinary tract infection
☐ Pyelonephritis

Post pancreatic surgery complications

☐ Postoperative pancreatic fistula A
☐ Postoperative pancreatic fistula B
☐ Postoperative pancreatic fistula C
☐ Delayed gastric emptying

Central nervous system complications

☐ Seizure
☐ Postoperative delirium
☐ Psychoorganic syndrome
☐ Stroke
☐ Intracranial hemorrhage

Post vascular surgery complications

☐ Occlusion
☐ Vessel/Bypass occlusion
☐ Bypass infection
☐ Lymphatic fistula

Cardiovascular complications

☐ Arterial occlusion
☐ Venous thrombosis
☐ Decompensated heart failure
☐ Myocardial infarction
☐ Angina pectoris
☐ Arrythmia

Infections

☐ Sepsis
☐ Candidiasis
☐ Systemic mycosis
☐ Viral infection
☐ Bacterial infection
☐ Clostridioides difficile infection

Admission

☐ Planned (re-) admission
☐ Emergency admission
☐ Transfer
☐ Readmission due to complications

Complications

Medical ☐ Yes ☐ No
Organizational ☐ Yes ☐ No
☐ Delayed diagnostics
☐ Intensive care unit capacity
☐ Operating room capacity
☐ Normal ward capacity
Others ______

Surgical site infections (SSI)

☐ SSI 1
☐ SSI 2
☐ SSI 3
☐ Fascia dehiscence

Bleeding complications

☐ Intraoperative bleeding
☐ Postoperative bleeding
☐ Hematoma
☐ Perioperative coagulopathy

Post-transplant complications

☐ Rejection
☐ Delayed graft function
☐ Graft failure
☐ Vascular complications
☐ "Steal" syndrome
☐ Ischemic type biliary lesion
☐ Graft pancreatits
☐ Retransplantation
Others ______

Clavien-Dindo classification

☐ Grade I
☐ Grade II
☐ Grade IIIa
☐ Grade IIIb
☐ Grade IVa
☐ Grade IVb
☐ Grade V
Date and cause of death ______

Pulmonary Complications

☐ Pneumonia
☐ Pleural effusion
☐ Pulmonary artery embolism
☐ Pneumothorax
☐ Respiratory failure
☐ Emypema
☐ Lung edema
☐ Unplanned reintubation

Gastrointestinal Complications

☐ Paralysis
☐ Ischemia
☐ Perforation
☐ Fistula
☐ Unplanned stoma formation
☐ Mechanical ileus
☐ Anastomotic leakage
☐ Delayed gastric emptying
☐ Gastrointestinal bleeding
Others ______

Hepatobiliary Complications

☐ Post-interventional pancreatitis
☐ Intervention necessary
☐ Insufficieny of biliodigestive anastomosis
☐ Bile leakage
☐ Bilioma
☐ Liver abscess
☐ Injury of common bile duct
☐ Cystic duct stump insufficiency
☐ Liver insufficiency
☐ Liver failure
Others ______

Complication management

☐ Surgery
☐ Readmission to the intensive care unit
☐ Percutaneaus drainage
☐ Dialysis
☐ Endoscopy
☐ Angiography
☐ Gastric tube
☐ Chest tube
☐ Cardiopulmonary resuscitation
☐ Transfusion of blood products
Others ______

Supplementary Figure 1: Englisch translation of digital quality management document. QM documents are prospectively collected on the day of discharge and validated daily by a group of senior consultant surgeons. It includes the indication for surgical treatment (blue), complications which occurred during the in-patient stay (red) and the necessary procedure for complication management (green). Complications are classified either as medical or organizational. Medical complications are further classified into renal, post pancreatic surgery, central nervous system, post vascular surgery, cardiovascular, systemic, or surgical site infections, bleeding, post organ transplantation, pulmonary, gastrointestinal, and hepatobiliary complications. Finally, complications are classified according to Clavien-Dindo.
Abbreviations: Endoscopic retrograde cholangiopancreatography (ERCP), Percutaneous transhepatic bile duct drainage (PTCD)
